# Supplementary material for: Antibacterial Activity and Mechanisms of TroHepc2-22, a Derived Peptide of Hepcidin2 from Golden Pompano (Trachinotus ovatus)
Source: Int J Mol Sci. 2023 May 25;24(11):9251. doi: 10.3390/ijms24119251 (PMC10253267; doi:10.3390/ijms24119251)
Supplement: Supplementary file 1 [file ijms-24-09251-s001.zip › ijms-2339383-supplementary.pdf]

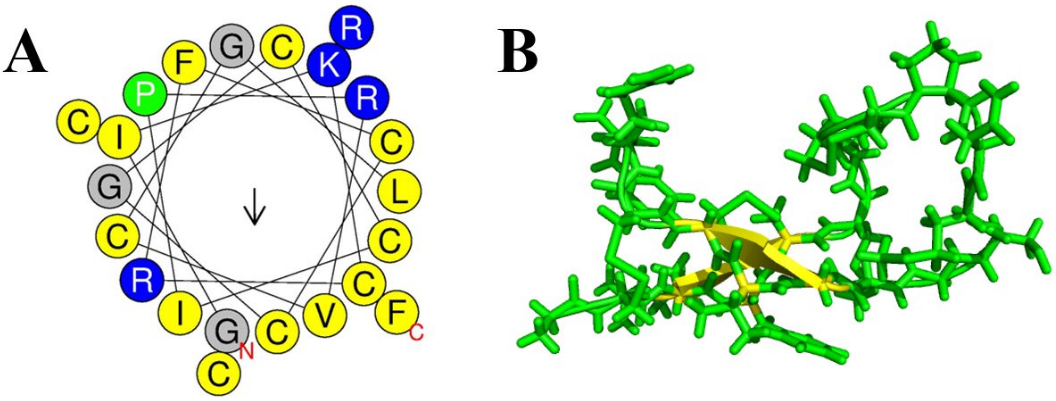

Figure S1. Predictive analysis and alignment of TroHepc2-22. (**A**) The helical wheel presentation of TroHepc2-22 predicted via the HeliQuest server. The arrow denoted the direction of hydrophobic face consisting of nonpolar residues; (**B**) Three-dimensional structure of TroHepc2-22 manifested by Pymol software. The loop was shaded in green and the sheet was shaded in yellow.
